# Buffalo Lithia Water

**Published:** 1881-04

**Authors:** 


BUFFALO LITHIA WATER.—We have spoken and written repeatedly in commendation of the Buffalo Lithia Water, and our views of its efficacy in several chronic diseases, are pretty generally known, particularly the water from spring No. 2; but we desire to add a few words regarding its ascertained value in Bright's Disease. A knowledge of its action in that disease thus far, would seem to warrant the belief that it would in many instances, at least, in its early stages, arrest it entirely; and in its more advanced stages, prove a decided comfort and palliative. We earnestly commend it as worthy of trial in Bright's Disease.

We learn that Prof. Christopher Johnston has recently resigned the Chair of Surgery in the University of Maryland, He succeeded the late Prof. N. R. Smith in this field, upon the resignation of the latter eleven years ago. Prof. L. McLane Tiffany, who now holds the Chair of Operative Surgery, will doubtless be elected to fill the vacancy.

